# Macronutrient Intake in Relation to Migraine and Non-Migraine Headaches

**DOI:** 10.3390/nu10091309

**Published:** 2018-09-15

**Authors:** Valentina A. Andreeva, Fabien Szabo de Edelenyi, Nathalie Druesne-Pecollo, Mathilde Touvier, Serge Hercberg, Pilar Galan

**Affiliations:** 1 Nutritional Epidemiology Research Group (EREN), University of Paris 13/INSERM U1153/INRA U1125/CNAM, COMUE Sorbonne Paris Cité, F-93017 Bobigny, France; f.szabo@eren.smbh.univ-paris13.fr (F.S.d.E.); n.pecollo@eren.smbh.univ-paris13.fr (N.D.‐P.); m.touvier@eren.smbh.univ-paris13.fr (M.T.); s.hercberg@eren.smbh.univ-paris13.fr (S.H.); p.galan@eren.smbh.univ-paris13.fr (P.G.); 2 Department of Public Health, Avicenne Hospital, F-93017 Bobigny, France

**Keywords:** macronutrients, dietary intake, migraine, headache, epidemiology

## Abstract

We investigated the association of mean daily macronutrient intake with migraine and non-migraine headaches. This cross-sectional study included 8042 men and 23,728 women from the ongoing population-based NutriNet-Santé e-cohort. Headache status was assessed via an online self-report questionnaire (2013–2016). Migraine was defined using established criteria and dietary macronutrient intake was estimated via ≥3 24 h dietary records. Mean daily intake (g/day) of carbohydrates (simple, complex, and total), protein, and fat (saturated fatty acids, monounsaturated fatty acids, polyunsaturated fatty acids, and total) were the main exposure variables. Adjusted gender-specific analysis of variance (ANOVA) models were fit. Presence of migraines was noted in 9.2% of men (mean age = 54.3 ± 13.3 years) and 25.7% of women (mean age = 49.6 ± 12.8 years). In adjusted models, we observed (1) somewhat lower protein (*p* < 0.02) and higher total fat (*p* < 0.01) intake among male migraineurs compared with males without headaches and those with non-migraine headaches; (2) somewhat higher total fat (*p* < 0.0001) and total carbohydrate intake (*p* < 0.05) among female migraineurs compared with females without headaches and those with non-migraine headaches. The findings, which provide preliminary support for modest gender-specific differences in macronutrient intake by migraine status, merit confirmation in different population-based settings, as well as longitudinally, and could help to inform future dietary interventions in headache prevention.

## 1. Introduction

Migraine is a primary headache disorder with recurrent, unilateral attacks of moderate to severe pain intensity and concurrent photophobia, phonophobia, and nausea [1,2]. Migraines predominantly affect reproductive-age adults and are at least twice as common among women as among men [3,4]. They are the seventh leading cause of years lived with disability [5], with a worldwide prevalence estimated at 11.6% [3].

The clinical expression of migraine might be influenced by dietary behaviours owing to their role in systemic inflammation, vasodilation, cerebral glucose metabolism, attack frequency and severity [6,7,8]. Diet-related factors (e.g., skipping meals, fasting, dehydration, alcohol use/withdrawal, and caffeine withdrawal) have been explored as migraine triggers, yet findings are inconclusive [9,10,11]. Likewise, there is a lack of consensus regarding the effects of dietary restrictions and elimination diets on migraines [11,12]. In turn, a recent narrative review reported preliminary support for the role of comprehensive diets (which regulate quantities of macronutrients and vitamins without excluding any food or ingredient) in the prevention of migraines and other headache disorders [8]. These authors reported that low-fat diets as well as diets featuring high omega-3/low omega-6 polyunsaturated fatty acids (PUFA) appeared to decrease the frequency of migraine attacks [8]. To our knowledge, only one study of the role of diet, including energy and macronutrient intake, in migraine has been carried out in a large population-based sample [13]. That study used National Health and Nutrition Examination Survey (NHANES) 1999–2004 data, only included women aged between 20 and 50 years, and reported no significant differences in dietary patterns by migraine status [13]. Given the paucity of research and gaps in knowledge, the objectives of the present study were to describe the sociodemographic profiles of adult men and women according to headache status, and to investigate the cross-sectional association of dietary macronutrient intake with non-migraine and migraine headaches.

## 2. Materials and Methods

### 2.1. NutriNet-Santé E-Cohort

We analysed data from the ongoing population-based NutriNet-Santé e-cohort launched in France in 2009 (www.etude-nutrinet-sante.fr). Its design and objectives have been described elsewhere [14]. Briefly, male and female volunteers aged ≥18 years with access to the Internet are recruited by means of traditional approaches (e.g., flyers, word of mouth) and periodic multimedia campaigns (on the television, in newspapers, and on the Internet). NutriNet-Santé was approved by the ethics committee of the French Institute for Health and Medical Research and by the National Commission on Informatics and Liberty. Eligible participants provide informed consent and an electronic signature. Afterwards, they complete a set of five baseline questionnaires: sociodemographics and lifestyle, physical activity, anthropometrics, health status, and diet. On a regular basis thereafter, they receive additional questionnaires on nutrition and/or health-related topics.

### 2.2. Migraine Assessment

As part of the follow-up, a questionnaire assessing headache history was administered between November 2013 and December 2016. It was made available on a rolling and non-mandatory basis to all active participants who had been part of the cohort for at least two and a half years. A total of 119,451 enrolees were solicited, of whom *n* = 37,125 returned a completed questionnaire and were thus eligible for the present analysis. 

The questionnaire was adapted from an existing tool validated for use with young French-speaking adults [15]. Participants were first asked whether at any time during their lifetime they had experienced a headache. Those who responded negatively represented the “no headache” category, while those who responded affirmatively were further queried about the frequency, severity, laterality, and duration of headache episodes, as well as potential experience of photophobia, phonophobia, and nausea. Definitions of migraine and non-migraine headaches were based on prior validation work [15] and the “probable migraine” category of *The International Classification of Headache Disorders*, 3rd ed. [2]. However, the available data did not allow for the precise distinction between migraines with and without aura [2], or between episodic (≤14 headache days/month) and chronic migraines (>15 headache days/month) [16].

### 2.3. Dietary Intake Assessment

Individual dietary intake information was obtained from at least three 24 h dietary records provided between enrolment in the cohort and administration of the migraine questionnaire. Generally, participants are asked twice a year to provide three self-report 24 h records on non-consecutive days (spread over a 2-week period), including two weekdays and one weekend day. The user-friendly web-based tool features a food/beverage browser, a search engine, a comprehensive user’s guide, and a built-in control system with visual cues and prompts designed to aid recall. For each food item or beverage that is consumed, participants are asked to provide detailed information about the quantity/portion size, recipe/seasoning, and corresponding settings (time and place). Portion sizes are estimated using validated photographs [17]. A published food composition table with >3300 different items is used to estimate macro- and micronutrient intake [18]. Mean macronutrient intake values were calculated for each participant from all available 24 h dietary records over the selected period. Under-reporting as regards energy intake was identified using Black’s method [19], which is based on an estimate of the person’s basal metabolic rate calculated via Schofield’s equations [20] and takes into account the person’s gender, age, height, weight, and physical activity level. Individuals with extreme or likely energy under-reporting were excluded from the analysis, as were those with fewer than three 24 h records [21,22].

### 2.4. Descriptive Characteristics

We used self-reported baseline data on sociodemographics (gender, age, marital status, education, and employment), lifestyle (physical activity, smoking), and health (weight, height, and family history of headaches). Body mass index (BMI) was calculated as the weight (in kilogrammes) divided by the squared height (in metres). Leisure-time physical activity was assessed with the International Physical Activity Questionnaire—Short Form and scoring followed the established protocol [23].

### 2.5. Statistical Analysis

**Figure 1:**
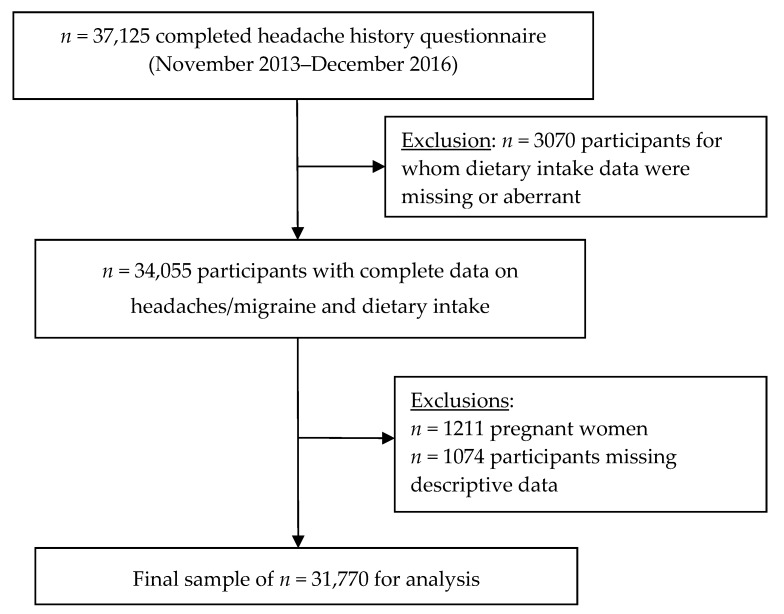
Participant selection flowchart.

From the sample of 37,125 individuals who completed the headache questionnaire, we excluded *n* = 2159 with missing or aberrant dietary intake data, *n* = 911 with <3 24 h dietary records, *n* = 1211 pregnant women (over the 9 months prior to, or the month following completion of the headache questionnaire), and *n* = 1074 individuals with missing descriptive data. A total of *n* = 1563 records were imputed using the mode of the respective variable (i.e., marital status, education, or employment). Thus, the final sample consisted of *n* = 31,770 individuals (Figure 1).

Chi-squared tests and ANOVA) were used for comparison of the descriptive characteristics across headache types. The principal outcome (dependent) measure was a three-category variable: “no headache” (reference category), “non-migraine headache”, and “migraine”, whereas exposure (independent) measures included mean dietary intake (g/day) of carbohydrates (simple, complex, and total), protein, and fat (saturated fatty acids, SFA; monounsaturated fatty acids, MUFA; PUFA; total). Cross-sectional associations stratified by gender were estimated via ANOVA adjusted for age, BMI, marital status, educational level, employment status, smoking status, physical activity level, and total energy intake. All tests were two-sided and *p* < 0.05 was considered statistically significant. The analyses were conducted with SAS software (version 9.4; SAS Institute Inc., Cary, NC, USA). 

## 3. Results

### 3.1. Sociodemographic Profiles by Headache Status

Among all NutriNet-Santé participants who completed the headache questionnaire, compared with those who were excluded (Figure 1: *n* = 5355), those who were included in the analysis (*n* = 31,770) were (1) somewhat older (52.4 ± 13.8 years vs 46.5 ± 14.8 years), (2) represented a higher percentage of men (25.3% vs 18.1%), (3) a somewhat higher percentage of individuals living alone (24.5% vs 21.5%), (4) and a higher percentage of retired individuals (34.2% vs 22.3%) (all *p* < 0.0001) (data not tabulated).

Descriptive characteristics by gender and headache status are summarised in Table 1. 

The sample consisted of *n* = 8042 men, of whom *n* = 737 (9.2%) had migraines (mean age = 54.3 ± 13.3 years), and *n* = 23,728 women, of whom *n* = 6097 (25.7%) had migraines (mean age = 49.6 ± 12.8 years). Among migraineurs, 43.6% of male participants and 32.7% of female participants were overweight (including obese participants). In both genders, individuals with non-migraine headaches were more likely to report a family history of headaches compared with individuals with migraines. Also, in both genders, the headache-free group had somewhat lower levels of vigorous physical activity compared with the two headache groups. There were higher percentages of female migraineurs who had never smoked (50.8% vs 41.4%) or who were smokers at the time of the study (11.9% vs 9.8%) compared with male migraineurs. Retired men and women were less likely to report migraines compared with those with a different employment status.

### 3.2. Association between Macronutrient Intake and Headache Status

The mean number of 24 h dietary records by gender and headache status varied from 15.1 to 16.7 (range: 3–27). Data on adjusted mean macronutrient intake by gender and headache status are shown in Table 2.

Among men, there was a slight decrease in mean protein intake across headache status (93.5 ± 0.2 in those without headaches; 92.6 ± 0.2 in those with non-migraine headaches; 91.9 ± 0.5 g/day in those with migraines; *p* < 0.02), whereas the reverse trend was observed regarding mean total fat intake (93.5 ± 0.2, 94.9 ± 0.2, and 95.5 ± 0.5 g/day, respectively; *p* < 0.01). The latter appeared driven by a slight increase in SFA and MUFA intake. In women, mean protein intake displayed non-significant associations with headache status. However, mean total fat intake appeared to increase slightly across headache status (76.6 ± 0.1, 77.2 ± 0.1, 77.4 ± 0.1 g/day, respectively; *p* < 0.0001), underscored by a slight increase in SFA and MUFA intake. In addition, mean total carbohydrate intake in women appeared somewhat higher in migraineurs compared with those in the “no headache” and “non-migraine headache” groups (184.1 ± 0.3 vs 183.4 ± 0.3 and 183.4 ± 0.2 g/day, respectively; *p* < 0.05). In men, no significant associations between mean carbohydrate intake (simple, complex, or total) and headache status were found.

## 4. Discussion

This large cross-sectional study provided some evidence of a significant albeit modest association between macronutrient intake and headaches in adults. Specifically, we observed somewhat lower protein and higher fat (SFA, MUFA, and total fat) intake among male migraineurs compared with males without headaches and those with non-migraine headaches. We also observed slightly higher total fat and total carbohydrate intake among female migraineurs compared with females without headaches and those with non-migraine headaches. Overall, protein and carbohydrate intake displayed gender-specific associations with headache status, whereas fat intake displayed relatively uniform associations across gender. Macronutrient intake in our sample varied by gender, as expected, being higher in men than in women. Recent national data revealed that in France, protein and carbohydrate intake is approximately 35% higher and fat intake is approximately 28% higher in men than in women [24]. However, it should be noted that the observed differences in macronutrient intake by headache status were very small in magnitude (despite their statistical significance given the large sample size) and were based on cross-sectional data. Hence, the results should be considered preliminary.

Our findings are consistent with a report of a positive correlation between baseline dietary fat intake and headache frequency in a small sample of migraineurs [25]. Results of various dietary interventions had also suggested that decreased dietary fat intake was associated with a decrease in headache frequency/intensity/duration among migraineurs [7,25,26]. Elevated blood lipids and free fatty acids have been associated with increased platelet aggregability, decreased serotonin, and heightened prostaglandin levels, all of which likely lead to vasodilatation preceding migraine occurrence [27]. In our analysis, the associations with dietary fat were driven by SFA and MUFA intake, whereas PUFA intake did not appear to play a role. Further, studies with middle-aged adults have revealed significant associations between total cholesterol and/or triglyceride concentrations and migraine [28,29], while research on the link between lipids, lipoprotein subfractions, and migraine has demonstrated that the worst lipid profile is underscored by the highest concentrations of triglyceride-rich lipoprotein cholesterol. It also associated cholesterol-rich remnants in both male and female migraineurs [30].

In our study, the association of migraine and mean carbohydrate intake was non-significant in men. In women, a significant albeit weak link between total carbohydrate intake and migraine was found. Evidence about the role of blood glucose in migraine suggested that glucose may be high in headaches, yet not specific to migraines [31]. It has also been hypothesised that minimising blood glucose fluctuations might help prevent migraines and other types of headaches [32]. However, the role of carbohydrate intake and/or glucose concentration in migraine might be nuanced by aura status. For example, a case-control study showed increased odds of migraines with aura among individuals in the top tertile of glucose concentrations [33]. The present data did not permit distinction and measurement of migraines with and without aura. Generally, about two-thirds of the migraine prevalence pertains to migraines without aura [34,35].

Next, we found a slight decrease in mean protein intake across headache status among men, whereas among women that association was non-significant. Protein intake is rarely investigated in migraine and the present study could provide impetus for research in that area. The percentage of energy provided by protein intake observed in our study was very similar to that reported in a representative sample of French adults aged 18–79 years [24] and also in a representative U.S. sample [13].

These results are based on cross-sectional data and do not permit inference of causality. In our analysis, the choice was made to model headache status as the outcome (dependent variable), however, the associations with dietary intake might be complex and possibly not unidirectional. For example, food craving has been identified as a premonitory and resolution symptom of migraine attacks, especially among females [36,37]. Further, research with migraineurs showed that carbohydrate-rich foods constituted a specific craving and that such foods were well tolerated even during migraine attacks [38]. In turn, clinical evidence has suggested that the ketogenic diet (a high-fat, low-carbohydrate diet that stimulates lipid metabolism and ketone body synthesis) might be effective in migraine prophylaxis [39]. A recent clinical trial with overweight female migraineurs demonstrated favourable effects of the ketogenic diet on frequency of migraines, possibly at least partly due to an augmentation in mitochondrial energy metabolism and a reduction in neural inflammation [40].

Another limitation of the study was the fact that it used a select, possibly health- and nutrition-conscious sample of volunteers with complete data, and generalisability of the findings is thus limited. About a third of the solicited individuals responded to the headache history questionnaire. Also, a comparison with national Census figures showed significant sociodemographic differences between the general French population and NutriNet-Santé, especially regarding marital status and the proportions of women and relatively well-educated individuals [41]. Whereas selection bias is present in most epidemiological research relying on non-random volunteer samples, in this study it was likely mitigated given the focus on an exposure-outcome association rather than migraine risk prediction or prevalence estimation [42].

Strengths of this study included investigation of migraine and non-migraine headaches according to established criteria, use of a large number of 24 h dietary records providing good-quality dietary data, and use of a very large, sociodemographically and geographically heterogeneous sample of men and women. In fact, current data on migraines in men are scant [4]. The findings, which provided some evidence of modest gender-specific differences in macronutrient intake by migraine status, merit confirmation in different population-based settings, as well as longitudinally, and could help to inform future dietary interventions in primary headache prevention.

## Figures and Tables

**Table 1:** Descriptive characteristics of study participants by gender and headache status (NutriNet-Santé, *n* = 31,770).

	Men (*n*= 8042)							Women (*n* = 23,728)						
	No Headache (*n* = 4085)		Non-Migraine Headache (*n* = 3220)		Migraine (*n* = 737)		*p* ^a^	No Headache (*n* = 6850)		Non-Migraine Headache (*n* = 10,781)		Migraine (*n* = 6097)		*p* ^a^
Age, year							*<0.0001*							*<0.0001*
18–39 year	478	(11.7)	496	(15.4)	118	(16.0)		1313	(19.2)	2853	(26.5)	1470	(24.1)	
≥40 year	3607	(88.3)	2724	(84.6)	619	(84.0)		5537	(80.8)	7928	(73.5)	4627	(75.9)	
Mean (SD)	59.3	(13.5)	55.3	(13.4)	54.3	(13.3)	*<0.0001*	53.9	(13.8)	49.6	(13.3)	49.6	(12.8)	*<0.0001*
Body Mass Index, kg/m^2^							*0.07*							*<0.0001*
Normal weight (<25.0)	2218	(54.3)	1696	(52.7)	416	(56.4)		4968	(72.5)	7626	(70.7)	4100	(67.2)	
Overweight (25.0–29.9)	1505	(36.8)	1186	(36.8)	249	(33.8)		1348	(19.7)	2162	(20.1)	1312	(21.5)	
Obese (≥30.0)	362	(8.9)	338	(10.5)	72	(9.8)		534	(7.8)	993	(9.2)	685	(11.2)	
Mean (SD)	25.2	(3.6)	25.3	(3.8)	25.2	(4.2)	*0.33*	23.5	(4.3)	23.8	(4.6)	24.0	(4.9)	*<0.0001*
Marital status							*0.09*							*0.09*
Married/cohabiting	3409	(83.5)	2626	(81.6)	602	(81.7)		4944	(72.2)	7915	(73.4)	4498	(73.8)	
Living alone (single/divorced/ widowed)	676	(16.5)	594	(18.4)	135	(18.3)		1906	(27.8)	2866	(26.6)	1599	(26.2)	
Educational level							*<0.0001*							*<0.0001*
Less than high school	1070	(26.2)	690	(21.4)	160	(21.7)		1328	(19.4)	1715	(15.9)	966	(15.8)	
High school diploma or equivalent	496	(12.1)	370	(11.5)	79	(10.7)		1015	(14.8)	1421	(13.2)	914	(15.0)	
Post-secondary education	2519	(61.7)	2160	(67.1)	498	(67.6)		4507	(65.8)	7645	(70.9)	4217	(69.2)	
Employment status							*<0.0001*							*<0.0001*
Employed, self-employed	1541	(37.7)	1637	(50.8)	384	(52.1)		3459	(50.5)	6651	(61.7)	3713	(60.9)	
Homemaker/disabled/unemployed	178	(4.4)	189	(5.9)	58	(7.9)		744	(10.9)	1400	(13.0)	949	(15.6)	
Retired	2366	(57.9)	1394	(43.3)	295	(40.0)		2647	(38.6)	2730	(25.3)	1435	(23.5)	
Physical activity ^b^							*<0.0001*							*<0.0001*
Low	2000	(49.0)	1340	(41.6)	307	(41.7)		2554	(37.3)	3391	(31.5)	2031	(33.3)	
Moderate	1332	(32.6)	1208	(37.5)	270	(36.6)		2846	(41.5)	4673	(43.3)	2563	(42.0)	
Vigorous	753	(18.4)	672	(20.9)	160	(21.7)		1450	(21.2)	2717	(25.2)	1503	(24.7)	
Smoking status							*0.38*							*<0.0001*
Never	1671	(40.9)	1344	(41.7)	305	(41.4)		3836	(56.0)	5583	(51.8)	3096	(50.8)	
Former	2051	(50.2)	1554	(48.3)	360	(48.8)		2309	(33.7)	3941	(36.6)	2278	(37.4)	
Current	363	(8.9)	322	(10.0)	72	(9.8)		705	(10.3)	1257	(11.7)	723	(11.9)	
Family history of headache ^c^			1626	(50.5)	279	(37.9)	*<0.0001*			5168	(47.9)	2179	(35.7)	*<0.0001*

Values refer to numbers (%) except when noted otherwise. SD, standard deviation. ^a^ *p*-values obtained from chi-squared tests or ANOVA, as appropriate. ^b^ Assessed with the International Physical Activity Questionnaire—Short Form; scoring followed established protocol. ^c^ Only individuals reporting non-migraine or migraine headaches were asked about family history.

**Table 2:** Mean macronutrient intake by gender and headache status (NutriNet-Santé, *n* = 31,770).

	Men (*n* = 8042)							Women (*n* = 23,728)						
	No Headache (*n* = 4085)		Non-Migraine Headache (*n* = 3220)		Migraine (*n* = 737)		*p*	No Headache (*n* = 6850)		Non-Migraine Headache (*n* = 10,781)		Migraine (*n* = 6097)		*p*
Number of 24-h records, mean (SD)	16.7	(6.9)	16.3	(6.9)	15.8	(7.2)	*0.002*	16.1	(6.8)	15.8	(6.8)	15.1	(6.8)	*<0.0001*
Total daily energy, kcal, mean (SD)	2254	(436.3)	2280	(429.3)	2245	(433.9)	*0.02*	1770	(332.6)	1789	(335.5)	1755	(339.5)	*<0.0001*
*%* total carbohydrates	41.1	(6.4)	41.1	(6.2)	41.1	(5.8)	0.99	41.3	(5.9)	41.3	(5.7)	41.5	(5.7)	*0.20*
*%* simple carbohydrates	18.2	(4.9)	18.3	(4.7)	18.3	(4.7)	*0.60*	20.0	(4.5)	19.9	(4.5)	19.9	(4.6)	*0.56*
*%* protein	16.7	(2.5)	16.5	(2.6)	16.5	(2.5)	*0.001*	17.0	(3.0)	16.9	(3.0)	17.1	(3.2)	*<0.0001*
*%* fat	37.0	(5.1)	37.6	(5.0)	37.9	(4.8)	*<0.0001*	38.7	(4.9)	39.0	(4.9)	39.0	(4.9)	*<0.0001*
Dietary intake, g/day, mean (SD)														
Total carbohydrates	232.3	(0.6)	232.3	(0.6)	232.5	(1.3)	*0.75*	183.4	(0.3)	183.4	(0.2)	184.1	(0.3)	*0.046*
Simple carbohydrates	103.0	(0.4)	103.6	(0.5)	103.7	(1.0)	*0.44*	88.6	(0.2)	88.3	(0.2)	88.4	(0.3)	*0.53*
Complex carbohydrates	129.3	(0.4)	128.7	(0.5)	128.9	(1.0)	*0.96*	94.8	(0.2)	95.1	(0.2)	95.7	(0.3)	*0.13*
Protein	93.5	(0.2)	92.6	(0.2)	91.9	(0.5)	*0.018*	74.6	(0.1)	74.2	(0.1)	74.4	(0.2)	*0.97*
Total fat	93.5	(0.2)	94.9	(0.2)	95.5	(0.5)	*0.008*	76.6	(0.1)	77.2	(0.1)	77.4	(0.1)	*<0.0001*
Saturated fatty acids (SFA)	38.3	(0.1)	39.0	(0.1)	39.7	(0.3)	*0.006*	31.2	(0.1)	31.8	(0.1)	31.8	(0.1)	*<0.0001*
Monounsaturated fatty acids (MUFA)	34.8	(0.1)	35.5	(0.1)	35.7	(0.2)	*0.028*	28.8	(0.1)	29.0	(0.1)	29.1	(0.1)	*0.001*
Polyunsaturated fatty acids (PUFA)	13.4	(0.1)	13.4	(0.1)	13.2	(0.1)	*0.51*	10.9	(0.0)	10.7	(0.0)	10.8	(0.0)	*0.077*

Values refer to mean (standard deviation, SD) except when noted otherwise. Analysis of variance (ANOVA, *F* test) adjusted for age, body mass index (BMI), marital status, educational level, employment status, smoking status, physical activity level, and total energy intake.

## References

[1] World Health Organization (2016). Headache Disorders Fact Sheet No. 277.

[2] Headache Classification Committee of the International Headache Society (IHS) (2018). Headache Classification Committee of the International Headache Society (IHS) The International Classification of Headache Disorders, 3rd edition. Cephalalgia.

[3] Woldeamanuel Y.W., Cowan R.P. (2017). Migraine affects 1 in 10 people worldwide featuring recent rise: A systematic review and meta-analysis of community-based studies involving 6 million participants. J. Neurol. Sci..

[4] Vetvik K.G., MacGregor E.A. (2017). Sex differences in the epidemiology, clinical features, and pathophysiology of migraine. Lancet Neurol..

[5] GBD 2015 Disease and Injury Incidence and Prevalence Collaborators (2016). Global, regional, and national incidence, prevalence, and years lived with disability for 310 diseases and injuries, 1990–2015: A systematic analysis for the Global Burden of Disease Study 2015. Lancet.

[6] Finkel A.G., Yerry J.A., Mann J.D. (2013). Dietary considerations in migraine management: Does a consistent diet improve migraine?. Curr. Pain Headache Rep..

[7] Ferrara L.A., Pacioni D., Di Fronzo V., Russo B.F., Speranza E., Carlino V., Gargiulo F., Ferrara F. (2015). Low-lipid diet reduces frequency and severity of acute migraine attacks. Nutr. Metab. Cardiovasc. Dis..

[8] Martin V.T., Vij B. (2016). Diet and headache: Part 2. Headache.

[9] Rockett F.C., de Oliveira V.R., Castro K., Chaves M.L., Perla A.D.S., Perry I.D. (2012). Dietary aspects of migraine trigger factors. Nutr. Rev..

[10] Kelman L. (2007). The triggers or precipitants of the acute migraine attack. Cephalalgia.

[11] Holzhammer J., Wober C. (2006). Alimentary trigger factors that provoke migraine and tension-type headache. Schmerz.

[12] Finocchi C., Sivori G. (2012). Food as trigger and aggravating factor of migraine. Neurol. Sci..

[13] Evans E.W., Lipton R.B., Peterlin B.L., Raynor H.A., Thomas J.G., O’Leary K.C., Pavlovic J., Wing R.R., Bond D.S. (2015). Dietary intake patterns and diet quality in a nationally representative sample of women with and without severe headache or migraine. Headache.

[14] Hercberg S., Castetbon K., Czernichow S., Malon A., Mejean C., Kesse E., Touvier M., Galan P. (2010). The NutriNet-Santé study: a web-based prospective study on the relationship between nutrition and health and determinants of dietary patterns and nutritional status. BMC Public Health.

[15] Guichard E., Montagni I., Tzourio C., Kurth T. (2016). Association between headaches and tinnitus in young adults: Cross-sectional study. Headache.

[16] Bigal M. (2009). Migraine chronification-concept and risk factors. Discov. Med..

[17] Le Moullec N., Deheeger M., Preziosi P., Monteiro P., Valeix P., Rolland M., Potier De Courcy G., Christides J.P., Cherouvrier F., Galan P. (1996). Validation du manuel photos utilisé pour l’enquête alimentaire de l’étude SU.VI.MAX. Cah. Nutr. Diét..

[18] Etude NutriNet-Santé (2013). Table de Composition des Aliments.

[19] Black A.E. (2000). Critical evaluation of energy intake using the Goldberg cut-off for energy intake: Basal metabolic rate. A practical guide to its calculation, use and limitations. Int. J. Obes. Relat. Metab. Disord..

[20] Schofield W.N. (1985). Predicting basal metabolic rate, new standards and review of previous work. Hum. Nutr. Clin. Nutr..

[21] Touvier M., Kesse-Guyot E., Méjean C., Pollet C., Malon A., Castetbon K., Hercberg S. (2011). Comparison between an interactive web-based self-administered 24 h dietary record and an interview by a dietitian for large-scale epidemiological studies. Br. J. Nutr..

[22] Lassale C., Castetbon K., Laporte F., Deschamps V., Vernay M., Camilleri G.M., Faure P., Hercberg S., Galan P., Kesse-Guyot E. (2016). Correlations between fruit, vegetables, fish, vitamins, and fatty acids estimated by web-based nonconsecutive dietary records and respective biomarkers of nutritional status. J. Acad. Nutr. Diet..

[23] IPAQ Group IPAQ Scoring Protocol. www.ipaq.ki.se.

[24] Agence Nationale de Sécurité Sanitaire de l’Alimentation (ANSES) (2017). Étude Individuelle Nationale des Consommations Alimentaires 3 (INCA 3). Rapport D’expertise Collective.

[25] Bic Z., Blix G.G., Hopp H.P., Leslie F.M., Schell M.J. (1999). The influence of a low-fat diet on incidence and severity of migraine headaches. J. Womens Health Gend. Based Med..

[26] Bunner A.E., Agarwal U., Gonzales J.F., Valente F., Barnard N.D. (2014). Nutrition intervention for migraine: A randomized crossover trial. J. Headache Pain.

[27] Bic Z., Blix G.G., Hopp H.P., Leslie F.M. (1998). In search of the ideal treatment for migraine headache. Med Hypotheses.

[28] Janoska M., Chorazka K., Domitrz I. (2015). Migraine frequency and its association with dyslipidemia in women. Neurol. Neurochir. Pol..

[29] Monastero R., Pipia C., Cefalù A.B., Liveri E.T., Rosano R., Camarda R., Camarda C. (2008). Association between plasma lipid levels and migraine in subjects aged > or =50 years: Preliminary data from the Zabut Aging Project. Neurol. Sci..

[30] Goulart A.C., Lotufo P.A., Santos I.S., Bittencourt M.S., Santos R.D., Blaha M.J., Jones S., Toth P.P., Kulkarni K., Benseñor I.M. (2018). The relationship between migraine and lipid sub-fractions among individuals without cardiovascular disease: A cross-sectional evaluation in the Brazilian Longitudinal Study of Adult Health (ELSA-Brasil). Cephalalgia.

[31] Cavestro C., Rosatello A., Micca G., Ravotto M., Pia Marino M., Asteggiano G., Beghi E. (2007). Insulin metabolism is altered in migraineurs: A new pathogenic mechanism for migraine?. Headache.

[32] Hufnagl K.N., Peroutka S.J. (2002). Glucose regulation in headache: Implications for dietary management. Expert Rev. Neurother..

[33] Sacco S., Altobelli E., Ornello R., Ripa P., Pistoia F., Carolei A. (2014). Insulin resistance in migraineurs: Results from a case-control study. Cephalalgia.

[34] Le H., Tfelt-Hansen P., Skytthe A., Kyvik K.O., Olesen J. (2012). Increase in self-reported migraine prevalence in the Danish adult population: A prospective longitudinal population-based study. BMJ Open.

[35] Streel S., Donneau A.F., Hoge A., Albert A., Schoenen J., Guillaume M. (2015). One-year prevalence of migraine using a validated extended French version of the ID Migraine: A Belgian population-based study. Rev. Neurol..

[36] Schoonman G.G., Evers D.J., Terwindt G.M., van Dijk J.G., Ferrari M.D. (2006). The prevalence of premonitory symptoms in migraine: A questionnaire study in 461 patients. Cephalalgia.

[37] Quintela E., Castillo J., Munoz P., Pascual J. (2006). Premonitory and resolution symptoms in migraine: A prospective study in 100 unselected patients. Cephalalgia.

[38] Blau J.N. (1993). What some patients can eat during migraine attacks: Therapeutic and conceptual implications. Cephalalgia.

[39] Barbanti P., Fofi L., Aurilia C., Egeo G., Caprio M. (2017). Ketogenic diet in migraine: Rationale, findings and perspectives. Neurol. Sci..

[40] Di Lorenzo C., Coppola G., Sirianni G., Di Lorenzo G., Bracaglia M., Di Lenola D., Siracusano A., Rossi P., Pierelli F. (2015). Migraine improvement during short lasting ketogenesis: A proof-of-concept study. Eur. J. Neurol..

[41] Andreeva V.A., Salanave B., Castetbon K., Deschamps V., Vernay M., Kesse-Guyot E., Hercberg S. (2015). Comparison of the sociodemographic characteristics of the large NutriNet-Santé e-cohort with French Census data: The issue of volunteer bias revisited. J. Epidemiol. Community Health.

[42] Andreeva V.A., Fezeu L.K., Hercberg S., Galan P. (2018). Obesity and migraine: Effect modification by gender and perceived stress. Neuroepidemiology.

